# Effects of Dietary Inclusion of Seaweed, Heat Stress and Genetic Strain on Performance, Plasma Biochemical and Hematological Parameters in Laying Hens

**DOI:** 10.3390/ani10091570

**Published:** 2020-09-03

**Authors:** Shima Borzouie, Bruce M. Rathgeber, Cassie M. Stupart, Janice MacIsaac, Leslie A. MacLaren

**Affiliations:** Department of Animal Science and Aquaculture, Dalhousie University, Truro, NS B2N 5E3, Canada; borzouie@dal.ca (S.B.); brathgeber@dal.ca (B.M.R.); cassiestupart@dal.ca (C.M.S.); janice.macisaac@dal.ca (J.M.)

**Keywords:** plasma, blood biochemistry, hematology, performance, *Chondrus crispus*, *Ascophyllum nodosum*, prebiotics, seaweed, heat stress, genetic strain

## Abstract

**Simple Summary:**

Prebiotics such as seaweeds have been proposed as positive influencers on hen health and productivity, including their response to stressors such as heat. The present study was undertaken to evaluate the effects of 3% red seaweed *Chondrus crispus* and 0.5% brown seaweed *Ascophyllum nodosum* supplements on the heat stress responses in two genetic strains of laying hens, Lohmann LSL-Lite (White) and Lohmann Brown-Lite (Brown). The short-term seaweed diet had little effect on blood characteristics, but long-term feeding affected several blood chemistry values. The seaweed supplement reduced the feed intake and improved the feed/egg efficiency in the short term, although did not consistently affect the long-term performance. Heat stress affected the leukocyte count and some plasma chemistry parameters, particularly in the Brown layer strain. Our results showed better production, feed efficiency and resistance to heat stress in the White layer strain, with significant liver enzyme changes in alkaline phosphatase (ALP), alanine aminotransferase (ALT) and gamma-glutamyl transferase (GGT). More research on the biological effects of seaweed supplementation is necessary to better understand its impacts on health and performance.

**Abstract:**

This study was planned to investigate the effects of seaweed supplementation, genetic strain, heat stress and their interactions on laying hen performances, blood chemistry and hematology. In a short-term trial, laying hens of the two genetic lines Lohman LSL-Lite (White) and Lohman Brown-Lite (Brown) were supplemented with *Chondrus crispus* (CC) at 3% for 21 days, while a control group was not. In a long-term trial, the same two strains were assigned to control (0%), 3% red seaweed *Chondrus crispus* (CC) or 0.5% brown seaweed *Ascophyllum nodosum* (AN)-supplemented diets for 41 weeks, concluding with a four-week control or heat-stress period. The White hens displayed higher egg production and a lower feed/egg ratio. The short-term inclusion of CC significantly reduced the feed intake, weight gain and feed/egg ratio. The long-term seaweed intake affected the plasma albumin and gamma-glutamyl transferase (GGT) (*p* < 0.05), and there were significant strain-heat stress interactions; heat stress in the Brown birds was associated with reduced protein, globulin and glucose and increased cholesterol and GGT levels and higher heterophil-to-lymphocyte (H/L) ratios (*p* < 0.05) in response to heat stress (*p* < 0.05). In conclusion, a long-term seaweed supplementation affected the plasma protein and enzyme profiles, yet had little effect on hen leukocyte counts and the overall performance.

## 1. Introduction

Interest in the use of prebiotics for poultry production is high as the need for alternatives to conventional antibiotics to promote better animal health and welfare grows. The reported beneficial effects of prebiotic consumption include the growth of beneficial microbes, inhibition of pathogen colonization, reduction of cancer risk and inflammation, improved mineral absorption and a reduction of total cholesterol, as well as low-density lipoprotein cholesterol and triglycerides in blood serum [1,2]. Seaweed polysaccharides (alginate oligosaccharides and neoagarooligosaccharides) with prebiotic properties hold considerable interest as natural, low-cost and highly nutritive additives for poultry [2,3,4,5]. Seaweed can be considered as an excellent source of bioactive compounds and dietary fiber, with strong antioxidant, anticoagulant, antiviral, anti-inflammatory, anticancer and antidiabetic properties [6,7]. Studied species reportedly contain highly bioactive metabolites such as vitamins, minerals, carotenoids, polysaccharides, polyphenols and polyunsaturated fatty acids [8]. Red species such as *Chondrus crispus* are high in proteins, with an amino acid profile relatively high in glutamic acid, aspartic acid, glycine, alanine, arginine and proline [9].

We and others have identified seaweeds as a prebiotic source due to their low digestibility, fermentability and ability to enhance the growth of beneficial bacteria and reduce the population of pathogenic bacteria [2,3,10]. The supplementation of chickens’ basal diets with seaweed has been shown to enhance gut health, productivity and dressing percentage [10,11,12,13]. Although many studies on the beneficial effects of seaweed on chickens have focused on broilers [11,14,15], the inclusion of seaweed in the diets of laying hens showed improvements in antimicrobial resistance, egg quality, feed conversion ratio and overall gut health by altering chicken immunity [12,16]. Collectively, these studies support the consideration of seaweed primarily as a prebiotic, recognizing that many seaweeds also are high-quality protein supplements. It is expected that the use of seaweed in diets will continue to increase, although more work is needed to understand how long-term seaweed supplementations impact the blood metabolism and leukocyte function, as well as overall health and performance.

Chickens respond to heat stress by generating a combination of behavioral, biochemical, physiological and immunological changes. Heat stress causes significant decreases in chicken performance and productivity through reducing the feed intake, growth rate, egg production, egg quality and feed efficiency [17]. Heat stress can reduce the immune response of birds by suppressing the production of antibodies [18,19,20]. The exposure of chickens to heat can also increase plasma corticosterone and decrease mineral levels, as well as cause a reduction in the activity and performance of lymphoid organs and total leukocytic count [21,22]. Examining the heat blood parameters and leukocyte numbers may provide useful clinical indicators of how birds respond to prebiotic supplements and heat stress and their interactions in long-term production settings. How these vary with genetic strain is also of interest, since genetic backgrounds are expected to influence the resistance of laying hens to heat stress [23].

Thus, the present study was undertaken (1) to evaluate the effects of seaweed and thermal heat stress for two commercial laying hen lines on performance, hematological and blood biochemical determinations as variables to assess the health effects and (2) to evaluate the interaction effects of seaweed, heat stress and bird strain on the performance, hematological and biochemical parameters.

## 2. Materials and Methods

### 2.1. Birds, Housing and Diets

Two strains of commercial laying hens, Lohmann LSL-Lite (White) and Lohmann Brown-Lite (Brown), were housed at the Atlantic Poultry Research Centre of the Dalhousie Agricultural Campus in Truro, Nova Scotia, Canada. For the short-term trial, 50 White and 50 Brown laying hens at 55 weeks of age were supplemented with 0 or 3% red seaweed *Chondrus crispus* (CC) for 21 days prior to sampling using a completely randomized design with bird strain and seaweed as the factors (*n* = 100, 20 cages, 5 hens/cag and; 5 cages of each strain-seaweed treatment combination, so that cage is the experimental unit). The long-term trial utilized 240 hens at 31 weeks of age in a factorial design testing the effects of bird strain, seaweed supplementation and heat stress. White or Brown hens (*n* = 240, 48 cages, 5 hens/cage and 4 cages of each treatment combination) were assigned to 0% or 3% (wt/wt) CC or to 0.5% (wt/wt) brown seaweed *Ascophyllum nodosum* (AN) (Tasco^®^, Acadian Seaplants Ltd., Dartmouth, Nova Scotia, Canada) from weeks 31 to 72. At 68 weeks of age, two birds from each cage (per strain and per seaweed treatment) were transferred to mobile battery cage units in a separate room for a heat-stress evaluation, where temperatures gradually rose from 25 °C to 33 °C from 11 a.m. to 6 p.m. for 28 days. 

For all trials, the lighting program used was the continuous lighting program of 16-h light and 8-h darkness per day. Basal diets were formulated based on the commercial requirements for Lohmann LSL-Lite provided by Lohmann Tierzucht GmbH, Cuxhaven, Germany (Table 1). Diet components were adjusted for the major nutrient contributions of the seaweeds to ensure diets were isocaloric and isonitrogenous. The poultry research unit is compliant with the Chicken Farmers’ of Canada’s On-Farm Food Safety Assurance Program, and the use of both CC and AN were approved by the Canadian Food Inspection Agency. Birds were housed in conventional battery cages with ad libitum access to food and water in temperature-controlled rooms at 25 °C, except for the heat stress treatment, as indicated above. All experimental procedures were approved by the Dalhousie University Faculty of Agriculture Animal Care and Use Committee (Protocol number 2018-031; approved 31.5.2018) and carried out in accordance with the 3R principles and the Council of Animal Care Guidelines on the care and use of farm animals for research, testing and teaching [24].

### 2.2. Seaweed Preparation

Cultivated *Chondrus crispus* were grown on land in saltwater by Acadian Seaplants Limited, Dartmouth, NS, Canada. It was dried at room temperature for 48 h in an environmentally controlled room at the Atlantic Poultry Research Centre and manually turned every few hours to allow for uniform drying. Following drying, the seaweed was ground through a 0.4-mm screen of a micro Wiley mill (model 3; Arthur H. Thomas Co., Philadelphia, PA, USA) to be mixed into the diet. Tasco^®^, a commercially available prebiotic supplement for livestock and poultry, was also obtained from Acadian Seaplants Limited and was received harvested and solar-dried. For CC and AN, crude protein, crude fat, calcium, total phosphorus, potassium, magnesium, sodium, copper, manganese and zinc were analyzed at the Nova Scotia Department of Agriculture Analytical Lab, Truro, NS, Canada (Supplementary Table S1). The specific amino acid analyses were provided by Acadian Seaplants Limited. Analyses were conducted by Silliker Canada Co., Markham, ON, Canada (Supplementary Table S1).

### 2.3. Layer Performance

Eggs were collected daily at the same time at 10 a.m., and the hen-day egg production percentage was recorded throughout the experiment. The egg production (%) was calculated on a per cage basis over the entire trial period. The total eggs laid per cage was divided by the number of days, which was multiplied by the number of hens (hen-day) on trial. The resulting value was then multiplied by 100. Individual feed intake and body weight for each cage were recorded throughout the trials to ascertain the variation. Feed intake (grams of feed per bird per day) was reported by determining the difference between the total daily feed given to each cage with the feed refusals collected and weighted. Eggs were collected daily, and body weights were determined on the first and last days of the trial to measure the body weight gain by subtracting the final weight from the initial weight. The weight gain and feed/egg were calculated based on the data recorded. All birds remained healthy during the whole trial.

### 2.4. Collection of Blood Samples and Chemical Analysis

At the end of the trial (55 weeks of age in the short-term trial and 72 weeks of age in the long-term trial), one bird from each cage was euthanized, and blood samples were collected for analysis in Vacutainer tubes (Becton Dickinson, Franklin Lakes, NJ, USA) containing sodium heparin. Approximately 2 mL of blood plasma was isolated from birds by centrifugation at 2000× *g* at 4 °C for 10 min. The separated plasma was stored overnight at 4 °C and then analyzed at Diagnostic Services, Atlantic Veterinary College, University of Prince Edward Island (Charlottetown, PEI, Canada). The concentration of total plasma proteins, albumins, globulins, glucose, cholesterol with the activities of aspartate aminotransferase (AST), alanine aminotransferase (ALT), gamma-glutamyltransferase (GGT), alkaline phosphatase (ALP) and glutamate dehydrogenase (GLDH) in the blood plasma were determined using a Roche Cobas 6000 analyzer (Roche Diagnostics, Mannheim, Germany). Duplicate samples were submitted, and results were averaged for each bird.

### 2.5. Hematology Sample Preparation

Blood samples collected at euthanasia were used immediately to create smears on a standard microscope slide. The smeared specimens were air-dried at room temperature for 24 h before being fixed and stained by Fisher HEMA 3 Stat packTM (Fisher Diagnostics, Middle-town, VA, USA). Dried samples were dipped into Hema 3 solutions, including HEMA 3 fixative, eosinophilic staining solution (HEMA 3 solution I) and basophilic solution (HEMA 3 solution II), followed by dipping in distilled water five times in a row for 1 s each step. Stained blood smear samples were then stored at room temperature for white blood cell count. The white blood cell count was performed manually in all samples. Hematological values were examined for differences due to strain, seaweed level and heat stress using a three-way Analysis of Variance (ANOVA) by the general linear model (GLM) procedure of the SAS software version 9.4 (SAS Institute Inc., Cary, NC, USA).

### 2.6. Statistical Analyses

Each cage of five birds was considered as an experimental unit. A completely randomized design was used to analyze the effects of the strain, seaweed intake and heat stress and their interactions. Data were analyzed by three-way Analysis of Variance (ANOVA) using the general linear model (GLM) procedure of the SAS software version 9.4 (SAS Institute, Cary, NC, USA). Some data sets, including AST, ALT, GGT and GLDH, in both trials were log-transformed to convert to a normal distribution prior to ANOVA. The obtained results were subjected to statistical calculations of the least square mean and standard errors of the mean values. Significance was declared at *p* ≤ 0.05, and where effects were significant, Tukey’s test method was used to separate the means.

## 3. Results

### 3.1. Layer Production Performance

The hen production performance data for the short-term trial is presented in Table 2. Our data showed that the feed intake and weight gain were not significantly affected by the strain of bird (*p* > 0.05). However, the body weight of the Brown strain was clearly higher than that of the White strain (*p* < 0.05). The White hens displayed a higher egg production and lower feed/egg ratio for the entirety of the trial compared to the Brown birds (*p* < 0.05). The feed intake, weight gain and feed/egg were significantly lower (*p* < 0.05) in laying hens with a short-term dietary supplementation of 3% CC compared with control birds. However, the short-term inclusion of red seaweed in laying hen diets had no effect on egg production (*p* > 0.05). There was no significant interaction between the short-term dietary inclusion of the seaweeds and the strain of bird (*p* > 0.05). The long-term trial layer production performance data is reported elsewhere, but briefly, White hens displayed consistently higher egg production, lower body weight and improved feed conversion compared to Brown hens (*p* < 0.05) [25]. Long-term seaweed intake and heat stress did not have a notable effect on any production parameters [25].

### 3.2. Blood Plasma Biochemistry

**Figure 1:**
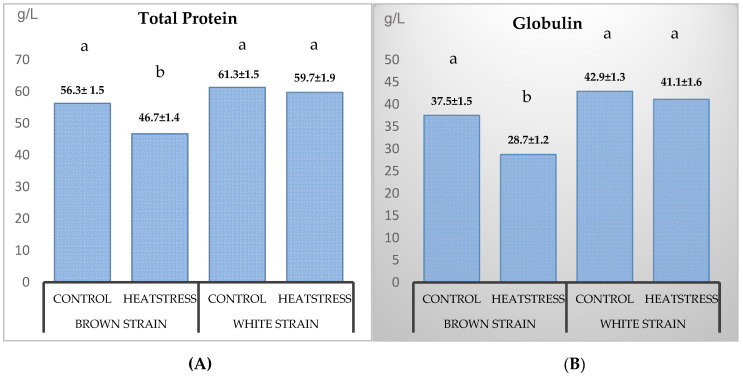

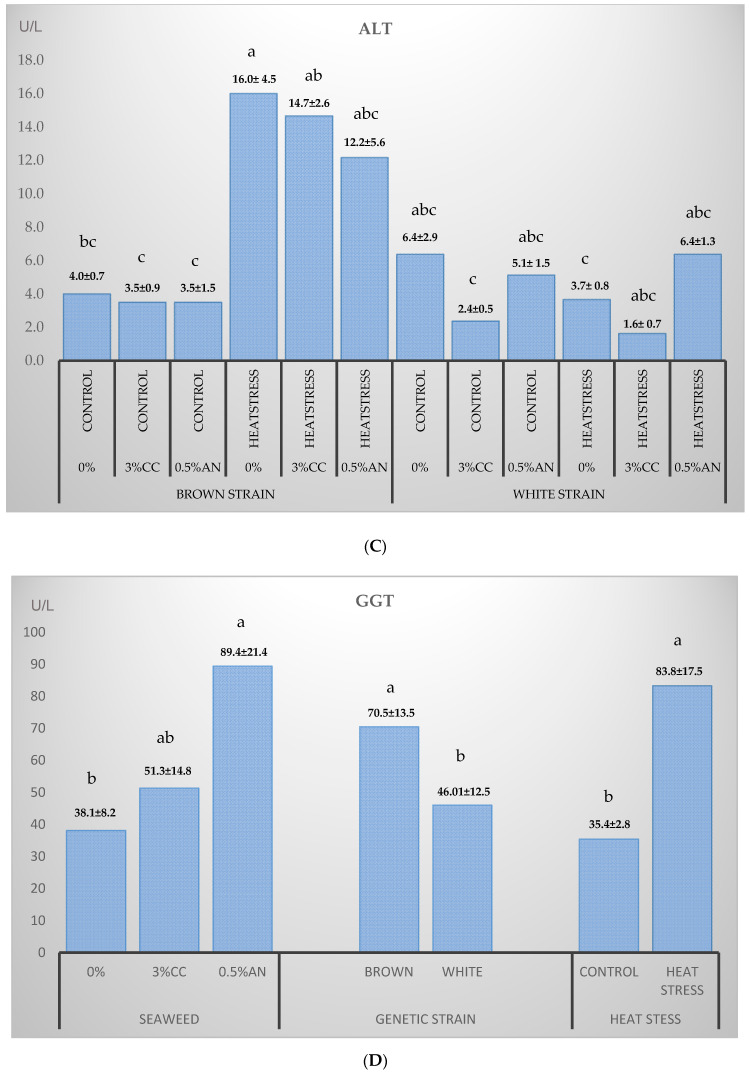
Effects of the genetic strain, long-term seaweed intake and heat stress on the total protein (**A**), globulin (**B**), alanine aminotransferase (ALT) (**C**) and gamma-glutamyl transferase (GGT) (**D**) in layer hens (*p*-value < 0.05). Values are least squares means ± standard error. a,b,c: Bars with different letters were different according to the Tukey means comparison test (*p* < 0.05). CC: *Chondrus crispus*. AN: *Ascophyllum nodosum*.

The effects of the experimental treatments on the blood biochemistry is summarized in Table 3, Table 4 and Table 5. The short-term seaweed supplementation elevated the globulin and protein levels in White birds but not in Brown. The remaining tested plasma biochemical parameters were not significantly (*p* > 0.05) influenced by the short-term seaweed supplementation (Table 3). The White birds fed with 0.5% AN seaweed had the highest globulin levels. Significant differences (*p* < 0.05) were noted between the long–term seaweed intake and control groups for albumin, ALT and GGT (Table 4). The statistical evaluation showed a significant increase of GGT in AN-supplemented hens versus control and CC-supplemented hens (*p* < 0.05; Figure 1D). Albumin and ALT levels were significantly reduced due to CC intake, while the Tasco^®^ (AN) diet caused an elevation of their levels (*p* < 0.05). We noticed an increase of AST and decrease of GLDH in seaweed-supplemented birds; however, the observed differences were not significant (*p* > 0.05).

Interestingly, in the short-term trial, the two strains of bird had similar enzyme profiles. However, the strain effect was more dominant in the long-term trial and expressed more, since significant differences in the biochemistry parameters were found between the two strains for the total protein, globulin, ALP, ALT and GGT (*p* < 0.05) (Table 4). These parameters were higher in the White strain, except for ALT and GGT, which were higher in the Brown strain (Figure 1). The White hen strain had higher plasma protein and globulin levels in both trials. As Table 5 demonstrates, the heat stress was associated with reduced protein, globulin and glucose and increased cholesterol and GGT levels (*p* < 0.05). There was, however, a significant interaction between bird strain and heat stress on the total protein, globulin, glucose, cholesterol and GLDH (*p* < 0.05). This interaction was also observed between the bird strain and seaweed for the effects on cholesterol (*p* < 0.05). Among the blood parameters studied, GGT was affected the most by the heat-stress treatment. As shown in Table 5, the only instance that the glucose level increased in the White strain was when both the heat stress and Tasco were applied, and a decrease in the GGT level happened only when both the 3% CC and heat stress were applied. Seaweed did not affect a heat stress-associated blood response. 

### 3.3. Hematology Analysis

**Figure 2:**
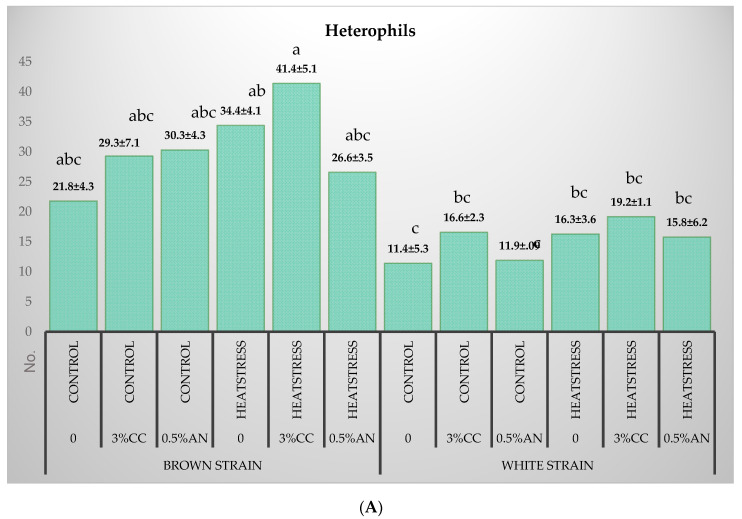

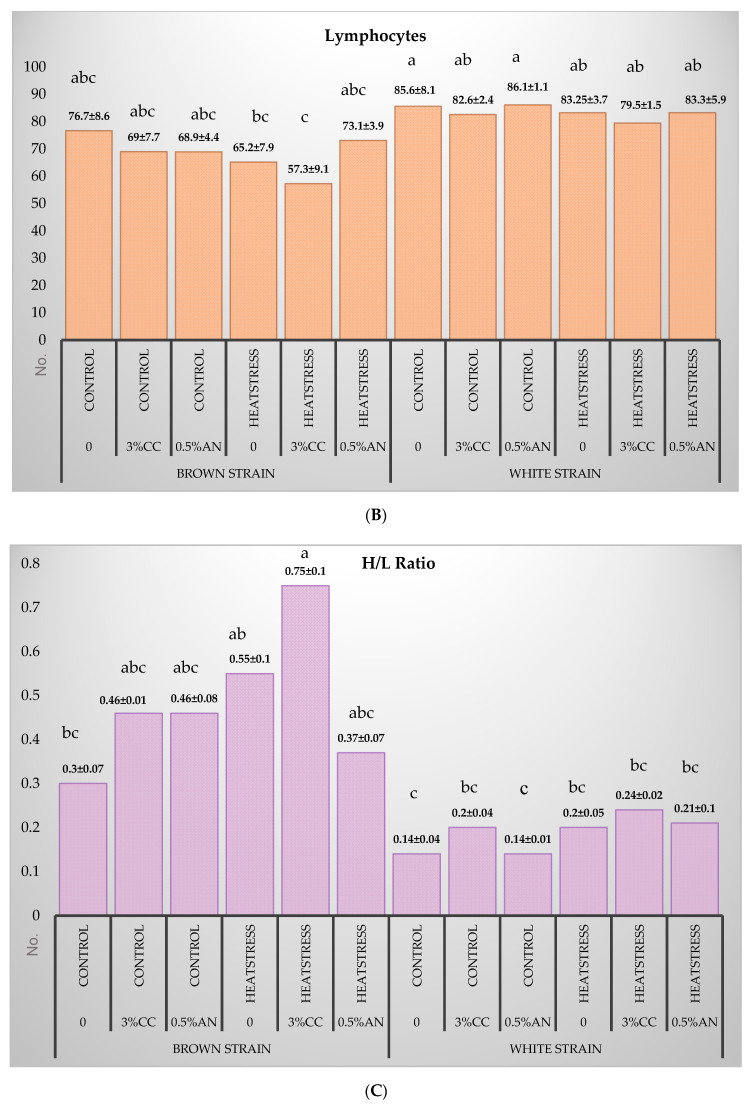
Heterophil number (**A**), lymphocyte number (**B**) and Heterophil/lymphocyte ratio (**C**) in White and Brown layer hens subjected to 4-week heat stress following the long-term feeding of seaweed supplements (means ± standard error). a,b,c: Bars with different letters were different according to the Tukey means comparison test (*p* < 0.05). CC: *Chondrus crispus*. AN: *Ascophyllum nodosum*.

The effects of three different treatments on the differential white blood cell counts and heterophil-to-lymphocyte ratio (H/L) were examined in this study. Basophils, eosinophils and monocytes accounted for less than 1% of the total leukocytes, roughly 23% for heterophils and about 76% for lymphocytes. The white blood cell count was not statistically associated with the seaweed intake, with no interactions with other treatments (*p* > 0.05). However, there was a significant effect of the strain on the values of the heterophil and lymphocyte counts, as the Brown strain had higher heterophil and lower lymphocyte counts and a higher H/L ratio (*p* < 0.05) (Figure 2). Furthermore, the blood eosinophil counts were associated with heat stress. After heat stress, the eosinophil number in Brown hens reduced from 0.51 to 0.27 (eosinophils/100 leukocytes) and, in White hens, from 0.83 to 0.17 (*p* < 0.05). The basophil number was not affected by any treatments in the study (*p* > 0.05).

A significant interaction was detected between the heat stress and strain for effects on the H/L ratio (Figure 2C). The H/L ratio increased in response to the heat stress in Brown, but not White, hens independent of the seaweed treatment (*p* < 0.05).

## 4. Discussion

### 4.1. Layer Performance

Our study indicated significant effects of short-term seaweed intake on laying hen performances. The reduction in feed intake decreased the feed/egg ratio, but the egg production remained at consistent levels throughout the experiment. Birds fed seaweed lost an average of 14% of their body weight (about 130 g) during the 21-day trial. However, in the long-term trial, the feed intake, feed/egg and body weight were not affected over the long term by the seaweed levels [25] which may be associated with an improvement of gut health and/or adaptation to the change in palatability of the diets. Similarly, the 21-day supplementation of broilers with 0.3% and 0.4% CC led to an increased productive efficiency, with significant decreases in the feed intake and feed conversion ratio [26]. However, the body weights of broilers did not show significant differences among broilers at 1, 21 and 32 days with CC supplementation [26]. This was in accordance with another 30-day feeding trial with no significant effect on the feed intake or body weight of laying hens fed on the 0.5%, 1% and 2% CC diets [8]. Several researchers reported similar performance results with other seaweeds. Ventura et al. studied the effect of inclusion of *Ulva rigida* seaweed on chicken performance, which showed that, as the level of seaweed increased, the feed intake and body weight gain decreased [27]. This shows a good correlation with these results. Ross and Dominy also reported that blue-green algae, *Spirulina platensis*, does not affect the growth performance and egg production of layer chickens [28]. Another study demonstrated that a supplementation of prebiotics had no impact on the feed intake of broilers in a 42-day trial [29]. In other avian species, it was previously reported that the dietary supplementation of another red seaweed (*Polysiphonis SPP*) did not have any effect on the growth performance and carcass quality in ducks [30]. Conversely, there were some studies that reported the body weight gain, feed intake and feed conversion of broilers were increased upon a prebiotic intake [31,32].

### 4.2. Blood Biochemistry

The blood enzyme activity appears to be a suitable diagnostic method to evaluate the hepatic function and reflect the degree of hepatocellular damage and leakage, tissue damage or organ dysfunction in birds [33]. As such, liver damage can be indicated by analyzing the plasma levels of ALT, AST, ALP, GGT and GLDH as the most frequently used biomarkers in clinical practice [33,34]. The plasma levels of ALT and AST reflect the integrity of hepatocytes, while ALP and GGT have limited roles in the diagnosis of hepatic diseases in birds [33,35]. Among all the blood parameters, the AST level is considered as the most specific and sensitive enzyme for detecting avian liver and muscle damage [33], and its level was not modified in response to the supplementation with seaweed, heat stress or among strains. Generally, the seaweed intake did not significantly affect the plasma enzyme levels, although the CC supplementation was associated with numerically decreased levels of ALT enzyme activity in White layers. It may be that a larger-scale study might show a modest beneficial effect. Conversely, the AN supplementation was associated with elevated GGT levels. Overall, the enzyme values were within the ranges observed previously in layers [36].

The genetic strain and heat stress had more widespread impacts on these indicator enzymes; for example, both GLDH and ALT were increased in response to heat stress in Brown, but not White, birds, indicating genetic differences in heat-stress susceptibility. Regardless of the strain, the plasma GGT level was elevated, over three times higher than normal level, when heat stress was applied. A high blood GGT level can be associated with an inflammatory response or indicate avian liver and biliary compromises [37,38]. Overall, our results are consistent with previous works in broiler chickens and rats that showed that the liver is susceptible to oxidative stress during acute heat exposure in broiler chickens [39,40]. 

The blood serum protein can reflect changes in the condition of an animal associated with inflammation, liver or kidney damage, as well as other internal and external factors. That heat stress was associated with decreased protein and globulin levels in Brown, but not White, birds further illustrates the former’s increased sensitivity to heat stress. Genetic differences in heat-stress responses have been observed by others [23]. The elevated plasma cholesterol and decreased glucose levels observed in Brown hens in response to heat stress were not predictable based on the studies of others, but certainly, similar changes have been observed, as discussed by El Kholy and colleagues [41]. 

Similarly, Kulshreshtha et al. reported that serum concentrations of the total protein, glucose and AST of layer hens supplemented with CC seaweed meal for 28 days were close to those of the control group [12]. Al-Harthi and colleagues found no significant changes in the ALP contents of plasma in laying hens after a brown seaweed intake [42], and others found no significant differences in the globulin and AST when a brown seaweed treatment was administered in laying hens [43]. Evenni et al. and Rizk et al.’s findings indicated that albumin levels were elevated in layers fed diets containing brown seaweed, which is in agreement with our results [44,45]. Albumin is associated with the functionality of the liver [44]. Different results were observed in other animal species, as rats fed brown seaweed resulted in a significant decrease of AST [44].

### 4.3. Hematological Parameters

The H/L ratio can provide an indicator of stress in birds, reflecting changes in the cellular and humoral immune system responses. High H/L ratios have been associated with increased stress and mortality, as well as weaker antibody responses in broiler chickens [46]. There are limited leucocyte studies in farm animals fed various seaweed species [47,48], and very little is known about the effect of the inclusion of seaweed on the white blood cell count and H/L ratio when fed to laying hens. Our earlier study showed reduced total leukocyte counts at a 2% CC supplementation and elevated total leukocyte counts at a 4% CC supplementation, so perhaps the current outcome of no observed impact at a 3% CC on the neutrophil, heterocyte or lymphocyte counts is not surprising. Similarly, Kang et al. reported that a supplementation of green algae *Chlorella vulgaris* had no impact on the blood leukocyte counts of broiler chickens, including heterophils, lymphocytes, monocytes, eosinophils or basophils [49]. However, supplemental *Ascophyllum nodosum* tended to increase (*p* ≤ 0.10) the white blood cell counts, eosinophils and lymphocytes in lambs [47]. In contrast, a reduction in the total leukocyte counts was observed in mice following an intake of red seaweed *Gracilaria birdiae* [50]. The difference between our results and some reports can be mainly due to the different animal species or seaweed types.

Our results also confirmed that the level of neutrophils was not different (*p* > 0.05) among the seaweed dietary treatments and control birds, which was similar to Sotoudeh and Jafari’s results in rainbow trout fed with red seaweed [48]. In contrast to these results, Vetvicka and Oliveria and Archer et al.’s findings indicated significant increases in the blood neutrophils of pigs and lambs [47,51]. 

That heat stress increased the blood H/L ratio in Brown layer hens was consistent with our plasma biochemistry observations that the Brown strain was more susceptible to the adverse effects of heat stress than the White strain. An elevated H/L ratio is expected in stressed hens, including in heat-stressed birds, and is thought to be a consequence of acute inflammatory responses to infectious, as well as noninfectious, causes [22]. The type and intensity of the immune response depends on the environmental and genetic factors, as well as the physiological status of the animal. This increased H⁄L ratio correlated with increased numbers of heterophils and decreased numbers of lymphocytes has been reported as a reliable indicator of life-threatening stressors in chickens [52,53].

## 5. Conclusions

Overall, White laying hens displayed a higher egg production and feed efficiency and were less susceptible to the adverse effects of heat stress, as indicated by the production, plasma components and liver enzyme profiles and white blood cell data. A long-term supplementation with *Chondrus crispus* or *Ascophyllum nodosum* seaweed did not negatively affect the plasma health indicators. *Chondrus crispus* supplementation at 3% transiently increased the feed efficiency in the short term, providing further evidence of its suitability for use in layer hens.

## Figures and Tables

**Table 1:** Diet formulation (g/kg) and calculated composition (as the fed basis) of the laying diet phases.

	Phase 1			Phase 2			Phase 3		
Ingredients	0%	3% CC	0.5% AN	0%	3% CC	0.5% AN	0%	3% CC	0.5% AN
Ground Corn	532.56	497.47	524.5	538.45	503.37	530.47	549.13	513.17	541.13
Canola Meal	100	100	100	100	100	100	100	100	100
Wheat	100	100	100	100	100	100	100	100	100
Soybean Meal	143.59	136.64	144.28	135.57	126.62	136.26	123.35	116.42	124.05
Limestone	45.15	44.96	45.07	46.43	46.26	46.35	47.7	47.55	47.61
Shell Mix	22.58	22.49	22.53	23.23	23.13	23.17	23.85	23.77	23.81
Oyster Shell	22.58	22.49	22.53	23.21	23.13	23.17	23.85	23.77	23.81
Animal/Vegetable Fat	11.82	27.47	14.7	11.93	27.57	14.81	11.42	27.09	14.31
Dicalcium Phosphate	11.18	11.39	11.21	10.8	11.01	10.83	10.44	10.66	10.48
Vitamin/Mineral Premix ^1^	5	5	5	5	5	5	5	5	5
Salt	3.73	0.14	3.33	3.61	0.02	3.21	3.63	0.04	3.22
Mehionine Premix ^2^	1.8	1.91	1.75	1.77	1.88	1.72	1.62	1.74	1.58
Tasco	0	0	5	0	0	5	0	0	5
Ground *Chondrus crispus*	0	30	0	0	30	0	0	30	0
Calculated Composition (%)									
Metabolizable Energy (kCal/kg)	2800	2800	2800	2800	2800	2800	2800	2800	2800
Protein (%)	16.04	16.04	16.04	15.71	15.71	15.71	15.22	15.22	15.22
Calcium (%)	3.73	3.73	3.73	3.82	3.82	3.82	3.91	3.91	3.91
Available Phosphorus (%)	0.4	0.4	0.4	0.39	0.39	0.39	0.38	0.38	0.38
Sodium (%)	0.17	0.17	0.17	0.16	0.16	0.16	0.16	0.16	0.16

^1^ Vitamin and mineral mixture (g/kg of premix): vitamin A (retinol), 1.56 g; vitamin D3 (cholecalciferol), 480.00 g; vitamin E (dl-alpha tocopheryl acetate), 8.00 g; vitamin K (menadione sodium bisulphate), 1.80 g; thiamine, 0.40 g; riboflavin, 1.90 g; pantothenic acid (as calcium pantothenate), 3.20 g; biotin, 32.00 g; folic acid, 4.40 g; vitamin B12, 2.30 g; niacin, 6.16 g; pyridoxine, 0.80 g; manganous oxide, 23.40 g; zinc oxide, 22.22 g; copper sulphate, 20.00 g; selenium premix, 14.86 g; ethoxyquin, 16.66 g; ground corn, 46.66 g and limestone, 100 g. ^2^ Methionine premix is composed of 50% wheat middlings and 50% DL methionine. CC, *Chondrus crispus* and AN, *Ascophyllum nodosum*.

**Table 2:** Effects of strain and seaweed intake on the means and standard errors of layer hen performances in the short-term trial.

Parameters	Brown Strain		White Strain		SEM	*p*-Value Strain	*p*-Value Seaweed
	0% CC	3% CC	0% CC	3% CC			
Egg production (%)	85.1 ^b^	81.3 ^b^	88.2 ^a^	87.1 ^a^	0.16	0.002	NS ^1^
Feed Intake (g/bird/day)	95.1 ^a^	85.9 ^b^	90.6 ^ab^	87.1 ^b^	29.5	NS	<0.001
Feed/egg	108.0 ^a^	96.7 ^bc^	96.7 ^b^	95.2 ^c^	1.7	0.02	0.02
Body Weight (g)	2113.3 ^a^	2083.9 ^ab^	1798.4 ^b^	1743.6 ^b^	36.3	<0.001	NS
Weight gain (g)	98.2 ^ab^	−119 ^b^	135.8 ^a^	− 142.4 ^b^	42.8	NS	<0.001

^a,b,c^ Least squares means within the same row with different superscripts were different according to the Tukey means comparison test (*p* < 0.05). ^1^ NS: no significant differences detected (*p*-value > 0.05). Note: No significant interaction was observed between the treatments.

**Table 3:** Genetic strain and seaweed effects on the plasma chemistry (least squares means ± standard error) in the short-term trial.

Plasma Parameter	Units	Brown Strain		White Strain		*p*-Value Strain	*p*-Value Seaweed
		0% CC	3% CC	0% CC	3% CC		
Protein	g/L	52.9 ± 3.4 ^b^	52.3 ± 1.8 ^b^	54.3 ± 0.9 ^ab^	64.0 ± 3.1 ^a^	0.02	NS ^1^
Albumin	g/L	18.1 ± 0.7	19.3 ± 1.1	17.4 ± 0.6	18.1 ± 0.4	NS	NS
Globulin	g/L	34.8 ± 2.7 ^b^	33.0 ± 0.9 ^b^	36.9 ± 1.3 ^ab^	46.0 ± 3.5 ^a^	<0.01	NS
Glucose	mM/L	14.2 ± 0.3	13.7 ± 0.4	13.5 ± 0.3	13.2 ± 0.3	NS	NS
Chol ^2^	mM/L	1.1 ± 0.2	0.9 ± 0.07	0.8 ± 0.07	0.8 ± 0.08	NS	NS
ALP ^3^	U/L	246.9 ± 47.2	206.8 ± 49.2	226.5 ± 44.5	202.3 ± 27.6	NS	NS
AST ^4^	U/L	194.9 ± 19.1	167.8 ± 8.3	181.0 ± 4.9	227.2 ± 32.7	NS	NS
ALT ^5^	U/L	4.6 ± 2.8	3 ± 0.3	2.7 ± 0.5	2.8 ± 0.3	NS	NS
GGT ^6^	U/L	24.3 ± 3.1	32.9 ± 5.7	21.7 ± 1.9	22.3 ± 2.3	NS	NS
GLDH ^7^	U/L	2.6 ± 0.4	2.4 ± 1.2	3.2 ± 1.6	1.3 ± 0.2	NS	NS

^a,b^ Least squares means within same row with different superscripts were different according to the Tukey means comparison test (*p* < 0.05). ^1^ NS: no significant differences detected (*p*-value > 0.05). ^2^ Chol: Cholesterol, ^3^ ALP: alkaline phosphatase, ^4^ AST: aspartate aminotransferase, ^5^ ALT: alanine aminotransferase, ^6^ GGT: gamma-glutamyl transferase and ^7^ GLDH: glutamate dehydrogenase. Note: No significant interaction was observed between the treatments.

**Table 4:** *p*-Values from the ANOVA test results from the long-term trial, showing the effects of the genetic strain, seaweed intake and heat stress and their interaction effects on the plasma chemistry in layer hens.

Plasma Parameter	Units	*p*-Value Strain	*p*-Value Seaweed	*p* -Value Heat Stress	Strain × Seaweed	Seaweed × Heat Stress	Strain × Heat Stress	Strain × Seaweed × Heat Stress
Protein	g/L	<0.01	NS ^1^	<0.01	NS	NS	0.02	NS
Albumin	g/L	NS	0.04	NS ^1^	NS	NS	NS	NS
Globulin	g/L	<0.01	NS	<0.01	NS	NS	0.02	NS
Glucose	mM/L	NS	NS	<0.01	NS	NS	0.01	NS
Chol	mM/L	NS	NS	<0.01	0.01	NS	<0.01	NS
ALP	U/L	<0.01	NS	NS	NS	NS	NS	NS
AST	U/L	NS	NS	NS	NS	NS	NS	NS
ALT	U/L	0.01	NS	<0.01	NS	NS	<0.01	NS
GGT	U/L	0.02	0.02	0.04	NS	NS	NS	NS
GLDH	U/L	NS	NS	NS	NS	NS	<0.01	NS

^1^ NS: no significant differences detected (*p*-value > 0.05) Note: No significant interaction was observed between the strain and seaweed treatments, except for cholesterol. AST: Aspartate aminotransferase, ALT: Alanine aminotransferase, GGT: Gamma-glutamyltransferase, ALP: Alkaline phosphatase, GLDH: Glutamate dehydrogenase.

**Table 5:** Effects of the long-term seaweed intake and a 28-day heat stress on the plasma biochemical analytes in two genetic strains of layers. Values shown as least squares means ± standard error.

	0% CC		3% CC		0.5% AN	
Brown Strain						
Plasma Parameter	Control	Heat Stress	Control	Heat Stress	Control	Heat Stress
Protein	59.7 ± 1.4 ^abc^	45.2 ± 1.9 ^d^	53.5 ± 3.2 ^abcd^	47.4 ± 3.4 ^cd^	55.9 ± 2.2 ^abcd^	47.6 ± 2.5 ^bcd^
Albumin	19.5 ± 0.5	18.1 ± 0.4	17.5 ± 0.7	17.4 ± 1.1	19.5 ± 0.9	18.6 ± 0.4
Globulin	40.5 ± 1.1 ^abc^	27.1 ± 2.1 ^d^	35.7 ± 3.8 ^abcd^	30.0 ± 2.5 ^bcd^	36.4 ± 2.1 ^abcd^	29.0 ± 2.2 ^cd^
Glucose	14.1 ± 0.3 ^ab^	11.9 ± 0.6 ^b^	14.1 ± 0.3 ^ab^	12.5 ± 0.8 ^b^	14.4 ± 0.8 ^ab^	11.8 ± 0.4 ^ab^
Chol	1.4 ± 0.3 ^c^	3.7 ± 0.4 ^a^	1.7 ± 0.2 ^bc^	3.4± 0.5 ^ab^	2.4 ± 0.3 ^abc^	3.8 ± 0.6 ^a^
ALP	156.7 ± 31.6	190.5 ± 59.8	195.4 ± 39.6	191.6 ± 66.3	171.9 ± 18.1	131.7 ± 28.1
AST	169.2 ± 7.9	156.125 ± 10.5	153.6 ± 13.7	198.1 ± 36.9	218.5 ± 30.1	194.4 ± 12.2
ALT	4.0 ± 0.7 ^bc^	16.0 ± 4.5 ^a^	3.5 ± 0.9 ^c^	14.7 ± 2.6 ^ab^	3.5 ± 1.5 ^c^	12.2 ± 5.6 ^abc^
GGT	32.5 ± 1.2	65.6 ± 30.9	42.2 ± 9.5	115.5 ± 48.6	49.4 ± 7.6	133.8 ± 56.3
GLDH	2.7 ± 2.3 ^b^	26.6 ± 8.4 ^a^	1.1 ± 0.6 ^b^	10.7 ± 7.7 ^ab^	6.6 ± 3.1 ^ab^	9.7 ± 5.1 ^ab^
**White strain**						
Protein	59.5 ± 2.1 ^abc^	58.5 ± 2.1 ^abcd^	61.5 ± 3.5 ^ab^	58.4 ± 5.1 ^abcd^	62.9 ± 2.0 ^a^	62.4 ± 2.4 ^a^
Albumin	18.0 ± 0.4	18.6 ± 0.7	18.7 ± 0.5	17.2 ± 0.6	18.4 ± 0.9	20.0 ± 1.1
Globulin	41.5 ± 2.4 ^abc^	39.9 ± 1.6 ^abc^	42.7 ± 3.1 ^a^	41.1 ± 4.5 ^abc^	44.5 ± 1.7 ^a^	42.4 ± 1.8 ^ab^
Glucose	13.7 ± 0.5 ^ab^	12.5 ± 0.4 ^ab^	13.7 ± 0.3 ^ab^	12.7 ± 0.3 ^ab^	14.0± 0.6 ^ab^	14.8 ± 0.7 ^a^
Chol	2.8 ± 0.3a ^bc^	3.5 ± 0.3 ^ab^	3.4 ± 0.4 ^ab^	2.2 ± 0.1 ^abc^	1.6 ± 0.2 ^bc^	2.5 ± 0.6 ^abc^
ALP	347.6 ± 46.9	263.6 ± 47.8	218.6 ± 31.4	264.7 ± 55.9	233.8 ± 9.1	219.9 ± 27.6
AST	190.1 ± 14.9	161.4 ± 9.4	176.5 ± 11.7	225.6 ± 31.9	199.6 ± 13.5	202.9 ± 27.3
ALT	6.4 ± 2.9 ^abc^	3.7 ± 0.8 ^c^	2.4 ± 0.5 ^c^	1.6 ± 0.7 ^c^	5.1 ± 1.5 ^abc^	6.4 ± 1.3 ^abc^
GGT	26.1 ± 4.5	28.0 ± 4.9	26.9 ± 6.9	20.4± 0.2	35.0 ± 1.8	139.4 ± 59.5
GLDH	9.4 ± 7.2 ^ab^	1.7 ± 0.7 ^b^	2.8 ± 0.8 ^b^	2.5 ± 0.5 ^b^	7.5 ± 2.9 ^ab^	3.6 ± 1.9 ^b^

^a,b,c,d^ Least squares means within the same row with different superscripts were different according to the Tukey means comparison test (*p* < 0.05). AST: Aspartate aminotransferase, ALT: Alanine aminotransferase, GGT: Gamma-glutamyltransferase, ALP: Alkaline phosphatase, GLDH: Glutamate dehydrogenase.

## Supplementary Materials

The following are available online at https://www.mdpi.com/2076-2615/10/9/1570/s1: Table S1: Chemical composition of seaweeds, *Chondrus crispus* and *Ascophyllum nodosum*, in 100 g of fresh weight.

## Abbreviations

CC	*Chondrus crispus*
AN	*Ascophyllum nodosum*
H/L	Heterophil/lymphocyte
APRI	Institute of the Dalhousie Agricultural Campus
AST	Aspartate aminotransferase
ALT	Alanine aminotransferase
GGT	Gamma-glutamyltransferase
ALP	Alkaline phosphatase
GLDH	Glutamate dehydrogenase
ANOVA	Analysis of Variance
SAS	Statistical analysis system
