# Supplementary material for: Effects of Dietary Inclusion of Seaweed, Heat Stress and Genetic Strain on Performance, Plasma Biochemical and Hematological Parameters in Laying Hens
Source: Animals (Basel). 2020 Sep 3;10(9):1570. doi: 10.3390/ani10091570 (PMC7552200; doi:10.3390/ani10091570)
Supplement: Supplementary file 1 [file animals-10-01570-s001.pdf]

animals

MDPI

Supplementary Table 1. Chemical composition of seaweeds, *Chondrus crispus and Ascophyllum nodosum*, in 100 grams of fresh weight.

**Parameters**	*Chondrus crispus*	*Ascophyllum nodosum*
**Aspartic Acid (%)**	1.44	0.53
**Threonine (%)**	0.63	0.25
**Serine (%)**	0.67	0.27
**Glutamic Acid (%)**	1.78	0.71
**Glycine (%)**	0.72	0.30
**Alanine (%)**	0.84	0.34
**Valine (%)**	0.71	0.27
**Methionine (%)**	0.28	0.11
**Isoleucine (%)**	0.59	0.26
**Leucine (%)**	0.96	0.38
**Tyrosine (%)**	0.36	0.12
**Phenylalanine (%)**	0.84	0.24
**Lysine (%)**	0.81	0.30
**Histidine (%)**	0.22	0.07
**Arginine (%)**	0.94	0.22
**Proline (%)**	0.70	0.25
**Hydroxyproline (%)**	<0.01	ND
**Cystine (%)**	0.28	0.07
**Tryptophan (%)**	0.17	0.06
**Calcium (%)**	0.39	1.28
**Potassium (%)**	4.11	2.14
**Magnesium (%)**	0.72	0.81
**Phosphorous (%)**	0.27	0.09
**Sodium (%)**	4.65	3.12
**Copper (ppm)**	< 5.00	< 5.00
**Manganese (ppm)**	84.95	23.98
**Zin (ppm)**	14.11	44.64
**Crude Fat (%)**	0.11	2.46
**Crude protein (%)**	19.31	5.2
**Dry matter (%)**	92.54	87.29
